# The association between peroxisome proliferator-activated receptor Δ rs3777744, rs3798343, and rs6922548 and coronary artery disease

**DOI:** 10.1042/BSR20181510

**Published:** 2019-01-03

**Authors:** Jing Zhang, Xiu-ling Liu, Qiao-wei Jia, Chen-hui Zhao, Jie-yin Liu, Feng-hui An, Li-hua Li, Zhao-hong Chen, Lian-sheng Wang, Wen-zhu Ma, Zhi-jian Yang, En-zhi Jia

**Affiliations:** 1 Department of Cardiovascular Medicine, The First Affiliated Hospital of Nanjing Medical University, Nanjing 210029, Jiangsu Province, China; 2 Department of Cardiovascular Medicine, The Friendship Hospital of Ili Kazakh Autonomous Prefecture, Yining 835000, Xinjiang, China

**Keywords:** coronary artery disease, peroxisome proliferator-activated receptor delta, single nucleotide polymorphism

## Abstract

**Objective**: The aim of the present study is to investigate the association between the single nucleotide polymorphism (SNP) sites of peroxisome proliferator-activated receptor Δ (PPARD) and the risk of coronary artery disease (CAD). To this end, a prospective observational single-center study of the clinical data from 880 subjects in a Chinese population was conducted. **Methods**: A total of 880 subjects, including 609 CAD patients and 271 control subjects, were selected for the present study. All inpatients had 4 ml of venous blood drawn after 12 h of fasting, and then clinical tests were conducted to obtain the biochemical parameters. CAD patients and Controls were distinguished by coronary angiography. Statistical analysis was conducted with SPSS software (ver 16.0). **Results**: A significant association between the G-alleles of PPARD rs3777744 and rs3798343 and a decreased risk for CAD was found. Moreover, we found an interaction between high fasting high-density lipoprotein cholesterol (HDL-C) serum levels, low serum glucose levels and their genotypes, ultimately decreasing the risk of CAD. Haplotype analysis was conducted on the three SNP sites, rs3777744 and rs3798343 to form a block [r^2^ = 0.79, D′ = 0.99). The A-C haplotypes were associated with an increased risk of CAD (odds ratio (OR), 95% confidence interval (CI): 1.321 (1.060–1.647), *P*=0.013], and the G-G haplotypes were associated with a decreased risk [OR, 95% CI: 0.714 (0.567–0.849), *P*=0.004]. **Conclusions**: Our study indicates a significant association between the G-alleles of PPARD rs3777744 and rs3798343 and a decreased CAD risk. In addition, genotypes interact with high serum HDL-C levels and low serum glucose levels, resulting in decreased prevalence of CAD.

## Introduction

Cardiovascular diseases (CVD), particularly coronary artery disease (CAD), are typically caused by atherosclerosis (AS) and are a major cause of deaths worldwide [1]. CAD is mainly diagnosed by angiography, an invasive examination that can be harmful to patients [2]. Despite great developments in the diagnosis and treatment of CAD, early diagnosis remains a challenge in clinical practice. As a result, specific and sensitive biomarkers for inchoate detection of CAD are necessary.

Peroxisome proliferator-activated receptors (PPARs) are ligand-activated nuclear transcription factors, including PPAR α (PPARA), PPAR Δ (PPARD), and PPAR γ (PPARG) [3]. PPARs are highly expressed in all tissues, including the brain, thyroid, and heart (from NCBI). Binding to specific elements in the promoter region of target genes, PPARs regulate the metabolism of lipids and carbohydrates by activating a network of downstream genes [3]. PPAR activation (or inhibition) can modulate multiple important biological processes that regulate peroxisomal function, lipid oxidation, metabolism of xenobiotics, lipid synthesis, adipocyte differentiation, and insulin action [4,5]. Furthermore, emerging evidence suggests a pivotal role for PPARs in various basic vascular processes. PPARA, PPARD, and PPARG are all expressed in endothelial cells, which play a critical role in vascular biology. Not only do endothelial cells protect the inner lining of vessels, they also deliver oxygen to the local blood vessels of all tissues. In *in vitro* and *in vivo* models, PPARA and PPARG have a critical role in general anti-inflammatory, anti-proliferative, and anti-angiogenic processes [6]. PPARD agonists can increase the angiogenic capacity and vasculogenesis of endothelial progenitor cells (EPCs) [7]. Meanwhile, they can increase the levels of anti-inflammatory molecules in human endothelial cells which decreases the formation of atherosclerotic lesions [8]. In addition, as a result of PPARs being inhibited by pro-inflammatory cytokines, energy production in the myocardium decreased [9]. The mechanisms above suggest that PPARD plays an important role in CAD.

Recently, it has been shown that LncPPARD regulates the expression of neighboring protein-coding genes. PPARD, with its direct target genes *ADRP* and *ANGPTL4*, might serve as a novel biomarker for CAD, particularly when combined with risk factors [9,10], since the positive association between PPARD polymorphism rs2016520 and CAD had previously been reported [11]. In this study, three novel polymorphic variants located in the intron of PPARD, namely, rs3777744, rs3798343, and rs6922548, were selected to further investigate the genetic mechanisms by which PPARD impacts the prevalence of CAD.

## Methods

### Subjects

A total of 880 consecutively sampled subjects (645 males and 235 females), including 609 CAD patients and 271 control subjects, were selected for the study to investigate the relationship between long non-coding RNA PPARD and the prevalence of CAD. All participants underwent coronary angiography at the Friendship Hospital of Ili Kazakh Autonomous Prefecture in China from 1 March 2010 to 31 April 2015 and were divided into the CAD group and the control group. The exclusion criteria included subjects with spastic angina pectoris, new infectious processes (symptoms for less than 2 weeks), heart failure, adrenal dysfunction, and thyroid dysfunction. CAD was diagnosed in subjects with at least one major epicardial vessel with >50% stenosis, and controls were defined as all major epicardial vessels with <50% stenosis [12]. Coronary arteries were cannulated using either the Judkins technique [13] or through a radial artery approach with 6F catheters, and the operations were conducted by at least two experienced cardiologists. The study was approved by the ethics committee of the First Affiliated Hospital of Nanjing Medical University and the Friendship Hospital of Ili Kazakh Autonomous Prefecture in China. All subjects provided written informed consent.

### Laboratory measurements

All inpatients had 4 ml of venous blood drawn after 12 h of fasting upon which clinical tests were conducted to obtain the biochemical parameters. The selected serum biochemical indices were primarily related to blood glucose and serum lipids, including total cholesterol (TC, mmol/l), triglyceride (TG, mmol/l), fasting blood glucose (FBG, mmol/l), fasting high-density lipoprotein cholesterol (HDL-C, mmol/l), fasting low-density lipoprotein cholesterol (LDL-C, mmol/l), apolipoprotein A (Apo-A, g/l), and apolipoprotein B (Apo-B, g/l). All biochemical assays were conducted using enzymatic methods on an automated analyzer (AU 2700 Olympus, 1st Chemical Ltd, Japan). The intra-assay and inter-assay CVs were <5% [14].

### Primer design and single nucleotide polymorphism selection

The original gene sequence covering the target single nucleotide polymorphism (SNP) sites was obtained from the NCBI website (www.ncbi.nlm.nih.gov) and further processed on the My Agena website. The ensemble genome browser (www.ensembl.org) was used to screen the SNP sites in the 5-near, seed and 3-near regions of LncPPARD. The minor allele frequencies (MAF) value was accessed on the International Genome website (www.internationalgenome.org), and all MAF values for selected SNP sites were confirmed as more than 0.05. The primers, PCR, and single base extension of the primers were designed by AssayDesigner 3.1 software (Sequenom Inc., San Diego, CA, U.S.A.). The primers were compounded by the same professional biotechnology company. All primers were diluted according to the manufacturer’s user guide.

### DNA extraction

A DNA extraction kit [Axygen Biotechnology (Hangzhou) Limited, Hangzhou City, China] was used to extract the DNA from peripheral blood. Quality control was conducted using 1.25% agarose gel electrophoresis (AGE), and the OD values were detected with Nanodrop 2000 spectrophotometer (Thermo, Wilmington, DE, U.S.A.). All samples were transferred to 96-well plates and stored at −20°C until used.

### Polymorphism genotyping

Genetic polymorphisms were identified on the Agena MassARRAY system (Agena/Sequenom Inc., San Diego, CA, U.S.A.) according to manufacturer’s user guide. DNA samples were amplified with the standard PCR. The final MgCl_2_ concentration was 3.5 mM, 1.875 mM from the PCR buffer and 1.625 mM from the MgCl_2_. Four microliters of PCR master mix was added to each well of the 384-well plates and was mixed uniformly with 1 μl of template DNA (20 ng/μl). Microplate sealers were used to prevent evaporation during the reaction.

The products of PCR were treated with shrimp alkaline phosphatase (SAP) to remove free dNTPs. The environment and procedure of the SAP reaction was set as: 37°C, 20 min; 85°C, 5 min; 4°C, ∞. Subsequently, single base extension (SBE) was conducted. Two microliters of extend mix and 7 μl of SAP+PCR reagent were mixed uniformly in each well of the 384-well plates. Microplate sealers were used to prevent evaporation.

Resin purification was conducted, and the products were transferred into a 384-well spectroCHIP bioarray with the MassARRAY Nanodispenser RS 1000 (Agena, Inc.). The gene chip was analyzed with MALDI-TOF-MS (MassARRAY Analyzer 4.0, Agena, Inc.). The original data and genotyping figures were obtained with MassARRAY TYPER4.0 software (Agena, Inc.). The integrity and validity of the output were examined and submitted to professional technologists for further analysis.

### Statistical analysis

Statistical analysis was conducted with the Statistics Package for Social Sciences (Ver. 16.0, SPSS Incorporated, Chicago, IL, U.S.A.). The relation between clinical biomarkers, including FBG, serum lipid, and the prevalence of CAD was evaluated by Student’s *t* test and the Mann–Whitney test, while the association between the parameters and allele distributions of the SNP sites was evaluated with one-way ANOVA and the Kruskal–Wallis test. Chi-square tests were used to assess the relationship between categorical variables. Receiver operating characteristic (ROC) analysis and logistic regression analysis were conducted to identify the predictors for CAD prevalence. *P*<0.05 was considered significant in the two-tailed tests. Skewed data are presented as the median (interquartile range), normal data are presented as the mean ± S.D., and categorical data are presented as the absolute value. All results were examined by PLINK software (Ver.1.07, Shaun Purcell), similar to the statistical analysis conducted by SPSS.

## Results

### The basic characteristics of the participants

Altogether, 880 patients (609 CAD and 271 controls) suspected of CAD were recruited for the present study. All participants underwent coronary angiography for the diagnosis of CAD at the Friendship Hospital of Ili Kazakh Autonomous Prefecture from 1 March 2010 to 31 April 2015. As shown in Table 1, 609 subjects were placed into the CAD group, and the control group was composed of 271 subjects. Subjects that were elderly (*P*<0.001), male (*P*<0.001), or frequent smokers (*P*=0.038), and patients with high FBG levels (*P*<0.001) and low HDL-C (*P*=0.011) were more susceptible to CAD. Serum levels of TC, TG, LDL-C, Apo-A, Apo-B, and drinking status were comparable between the CAD and control groups.

**Table 1:** Baseline characteristics of the subjects

Variables	Cases (*n*=609)	Controls (*n*=271)	*P*-values
Age (years)	61 (53–70)	58 (49–66)	<0.001
Gender (male/female)	472/137	173/98	<0.001
FBG (mmol/l)	5.2 (4.65-6.17)	4.86 (4.54–5.33)	<0.001
TG (mmol/l)	1.8 (1.24–2.48)	1.71 (1.17–2.41)	0.188
TC (mmol/l)	4.64 (3.87–5.52)	4.59 (3.91–5.29)	0.318
HDL-C (mmol/l)	1.32 (1.1–1.62)	1.4 (1.18–1.68)	0.011
LDL-C (mmol/l)	2.77 (2.19–3.47)	2.69 (2.14–3.36)	0.103
Apo(A)	1.28 (1.14–1.45)	1.3 (1.18–1.49)	0.053
Apo(B)	0.92 ± 0.23	0.90 ± 0.21	0.182
Smoking status (yes/no)	291/318	109/162	0.038
Drinking status (yes/no)	100/509	39/232	0.446

Skewed data are summarized by 50th (25–75th) percentiles, normal data are summarized by the mean ± S.D., and binary variables by N1/N2. Smoking status, drinking status, gender, and genotype were examined by Chi-Square tests, the serum level of ApoB was examined by Independent Samples *t* tests, and the rest baseline characteristics were examined by Mann–Whitney tests.

### Chi-square analysis of the association between SNP sites and CAD risk

The distribution of the rs3798343 genotype is significantly different in the control and CAD groups (*P*=0.006). Conversely, the distribution of the rs3777744 (*P*=0.064) and rs6922548 (*P*=0.520) genotypes are comparable between the CAD and control groups (Table 2).

**Table 2:** The Chi-square tests on the association between SNP sites and CAD risk

	CAD (*n*=609)	Control (*n*=271)	Chi-Square	*P*-value
rs3777744 (AA/GA/GG)	333/228/48	126/116/29	5.494	0.064
rs3798343 (CC/CG/GG)	378/192/39	137/112/22	10.260	0.006
rs6922548 (AA/AG/GG)	554/54/1	252/19/0	1.307	0.520

### Logistic regression analysis of the association between SNP sites and CAD

To further investigate the relationship between the selected SNP sites and CAD, logistic regression analysis was conducted. The genotype distribution of PPARD polymorphism rs3777744 (A>G) is consistent with the Hardy–Weinberg Equilibrium in both the CAD (*P*=0.309) and control groups (*P*=0.766). The frequencies of allele A are 73.4 and 67.9% in the CAD and control groups, respectively. All genotypes (AA, *P*=0.065; AG, *P*=0.056, GG, *P*=0.069) had different distributions in the CAD and control groups, and the GG genotype distribution was significantly different between the CAD patients and the control subjects (adjusted odds ratio (AOR), 95% confidence interval (CI): 0.724, 0.532–0.985; P*=0.04) when the *P*-value is adjusted for FBG. The genotype distribution of rs3777744 conforms to the dominant model (P*=0.023), and the GG+GA genotype serves as a protective factor in the prevalence of CAD (AOR, 95% CI: 0.714, 0.534–0.954) (Table 3). The genotype distribution of SNP site rs3798343 (C>G) is consistent with the Hardy–Weinberg Equilibrium in the control group (*P*=0.894) but not in the CAD group (*P*=0.033). The frequencies of allele C are 77.83 and 71.22% in CAD and control groups, respectively. The CC and GG genotypes (CC, *P*=0.005; GG, *P*=0.001) were distributed differently in the CAD and control groups when the *P*-value was adjusted for FBG. The genotype distribution of rs3798343 conforms to the dominant model (P*=0.001), and the GG+GC genotype also serves as a protective factor for CAD (AOR, 95% CI: 0.617, 0.460–0.826) (Table 4). The genotype distribution of rs6922548 was comparable in the two groups. However, small mutations are contained in the subjects, which leads to uncertainty in results (Table 5).

**Table 3:** Logistic analysis on the association between SNP rs3777744 and CAD risk

rs3777744 (A>G)	CAD (*n*=609)	Control (*n*=271)	OR (95% CI)	*P*-value	AOR* (95% CI)	P* value
AA	333	126	1.000 (reference)	0.065	1.000 (reference)	0.072
GA	228	116	0.744 (0.549–1.007)	0.056	0.671 (0.403–1.116)	0.124
GG	48	29	0.626 (0.378**–**1.037)	0.069	0.724 (0.532–0.985)	0.040
Dominant model (GG+GA compared withAA)	276/333	145/126	0.72 (0.540–0.960)	0.025	0.714 (0.534–0.954)	0.023
Recessive model (GG compared with GA+AA)	48/561	29/242	0.714 (0.440–1.160)	0.173	0.772 (0.473–1.261)	0.301
Allele A frequency	894 (73.40%)	368 (67.90%)				
Allele G frequency	324 (26.60%)	174 (32.10%)				
HWE	0.309	0.766				

GG represents the homozygote of minor alleles, AG represents the heterozygote, AA represents the homozygote of major alleles. Abbreviations: HWE, *P*-value for Hardy–Weinberg Equilibrium test; OR, odds ratio.

* Adjusted for FBG.

**Table 4:** Logistic analysis on the association between SNP rs3798343 and CAD risk

rs3798343 (C>G)	CAD (*n*=609)	Control (*n*=271)	OR (95% CI)	*P*-value	AOR* (95% CI)	P* value
CC	378	137	1.000 (reference)	0.006	1.000 (reference)	0.005
CG	192	112	0.621 (0.458–0.842)	0.002	0.669 (0.381–1.176)	0.162
GG	39	22	0.642 (0.368–1.123)	0.120	0.606 (0.445–0.825)	0.001
Dominant model (GG+CG compared with CC)	231/378	134/137	0.625 (0.468–0.834)	0.001	0.617 (0.460–0.826)	0.001
Recessive model (GG compared with CG+CC)	39/570	22/249	0.774 (0.450–1.333)	0.356	0.813 (0.470–1.407)	0.460
Allele C frequency	948 (77.83%)	386 (71.22%)				
Allele G frequency	270 (22.17%)	156 (28.78%)				
HWE	0.033	0.894				

GG represents the homozygote of minor alleles, CG represents the heterozygote, CC represents the homozygote of major alleles. Abbreviations: HWE, *P-*value for Hardy-Weinberg Equilibrium test; OR, odds ratio.

* Adjusted for FBG.

**Table 5:** Logistic analysis on the association between SNP rs6922548 and CAD risk

rs6922548 (A>G)	CAD (*n*=609)	Control (*n*=271)	OR (95% CI)	*P-v*alue	AOR* (95% CI)	P* value
AA	554	252	1.000 (reference)		1.000 (reference)	
AG	54	19	1.293 (0.751–2.226)	0.354	1.040E9	1.000
GG	1	0	-	-	1.368 (0.791–2.365)	0.263
Dominant model (GG+AG compared with AA)	55/554	19/252	1.317 (0.765–2.265)	0.320	1.399 (0.810–2.416)	0.229
Recessive model (GG compared with AG+AA)	1/608	0/271	-	-	-	-
Allele A frequency	1162 (95.40%)	523 (96.49%)				
Allele G frequency	56 (4.60%)	19 (3.51%)				
HWE	0.791	0.550				

GG represents the homozygote of minor alleles, AG represents the heterozygote, AA represents the homozygote of major alleles. Abbreviations: HWE, *P*-value for Hardy–Weinberg Equilibrium test; OR, odds ratio.

* Adjusted for FBG.

### ROC curve analysis including the optimal cut-off value and the Youden index for predicting CAD prevalence

ROC curve analyses were conducted to predict the prevalence of CAD. The area under curve (AUC) is 0.586 for age (95% CI: 0.545-0.627, *P*<0.001); 0.615 for FBG (95% CI: 0.576–0.653, *P*<0.001), and 0.554 for HDL-C (95% CI: 0.514–0.594, *P*=0.011) (Table 6).

**Table 6:** ROC curve analyses including the optimal cut-off value and the Youden index for the predicting of CAD prevalence

Variables	Auc (95% CI)	*P*-value	Cut-off	Sensitivity	Specificity	Youden index
Age	0.586 (0.545–0.627)	<0.001	59.5	0.567	0.557	0.124
Fbg	0.615 (0.576–0.653)	<0.001	5.445	0.427	0.793	0.22
TG (mmol/l)	0.528 (0.486–0.570)	0.188	-	-	-	-
TC (mmol/l)	0.521 (0.481–0.561)	0.318	-	-	-	-
HDL-C (mmol/l)	0.554 (0.514–0.594)	0.011	1.325	0.609	0.502	0.111
LDL-C (mmol/l)	0.534 (0.493–0.575)	0.103	-	-	-	-
Apo (A)	0.541 (0.500–0.582)	0.053	-	-	-	-
Apo (B)	0.526 (0.485–0.566)	0.223	-	-	-	-

AUC, the closer it is to 0.5, the less predictive it is.

### Gene–environment interaction analysis for predicting CAD prevalence

As is shown in Table 7, AA genotype (odds ratio (OR), 95% CI: 1.698 (1.124–2.566), *P*=0.012) and advanced age (≥59.5 years) appear to be risk factors in the prevalence of CAD. Those with the AA genotype (OR, 95% CI: 4.767 (2.736–8.306), *P*<0.001) or GG+GA genotype (OR, 95% CI: 1.727 (1.121–2.659), *P*=0.013) of rs3777744 with high FBG (≥5.445) are more likely to develop CAD. In subjects with the GG+GA genotype, HDL-C (OR, 95% CI: 0.567 (0.363–0.886), *P*=0.013) and no drinking status (OR, 95% CI: 0.707 (0.518–0.965), *P*=0.029) serve as protective factors against CAD. In addition, females (AA: OR, 95% CI: 0.514 (0.327–0.808), *P*=0.004; GG+GA: OR, 95% CI: 0.384 (0.249–0.593), *P*<0.001) and those with high HDL-C (AA: OR, 95% CI: 0.514 (0.337–0.784), *P*=0.002; GG+GA: OR, 95% CI: 0.443 (0.290–0.677), *P*<0.001) subjects (HDL-C ≥ 1.325) of either AA genotype or GG+GA genotype are less susceptible to CAD. In the recessive model, advanced age (≥59.5 years) in either the GG genotype (OR, 95% CI: 3.375 (1.249–9.12), *P*=0.016) or the GA+AA genotype (OR, 95% CI: 2.839 (1.494–5.39), *P*=0.001) appears to be a risk factor for CAD. Patients with advanced age (≥59.5 years) (OR, 95% CI: 2.839 (1.494–5.39), *P*=0.001), high FBG (≥5.445) (OR, 95% CI: 3.588 (1.951–6.599) and smoking habits (OR, 95% CI: 2.309 (1.181–4.516), *P*=0.014) with the GA+AA genotype of rs3777744 are more susceptible to CAD.

**Table 7:** CAD incidence by interactions with environmental factors such as age, gender, smoking status, drinking status, the serum level of HDL-C and FBG

Characteristics	Dominant model of rs3777744 [OR (95% CI); *P-*value]		Recessive model of rs3777744 [OR (95% CI); *P*-value]	
	AA	GG+GA	GG	GA+AA
Age				
<59.5	1.000 (reference)	0.771 (0.516–1.152; *P*=0.204)	1.000 (reference)	1.869 (0.984–3.549; *P*=0.056)
≥59.5	1.698 (1.124–2.566; *P*=0.012)	1.194 (0.788–1.808; *P*=0.404)	3.375 (1.249–9.121; *P*=0.016)	2.839 (1.494–5.396; *P*=0.001)
Fbg				
<5.445	1.000 (reference)	0.851 (0.605–1.196; *P*=0.352)	1.000 (reference)	1.245 (0.712–2.177; *P*=0.443)
≥5.445	4.767 (2.736–8.306; *P*<0.001)	1.727 (1.121–2.659; *P*=0.013)	2.400 (0.771–7.468; *P*=0.131)	3.588 (1.951–6.599; *P*<0.001)
HDL-C				
<1.325	1.000 (reference)	0.567 (0.363–0.886; *P*=0.013)	1.000 (reference)	1.788 (0.888–3.600; *P*=0.104)
≥1.325	0.514 (0.337–0.784; *P*=0.002)	0.443 (0.290–0.677; *P*<0.001)	0.933 (0.371–2.347; *P*=0.883)	1.085 (0.545–2.160; *P*=0.816)
Gender				
Male	1.000 (reference)	0.733 (0.517–1.039; *P*=0.081)	1.000 (reference)	1.797 (0.998–3.237; *P*=0.051)
Female	0.514 (0.327–0.808; *P*=0.004)	0.384 (0.249–0.593; *P*<0.001)	1.111 (0.413–2.989; *P*=0.835)	0.850 (0.456–1.583; *P*=0.608)
Smoking status				
No	1.000 (reference)	0.741 (0.507–1.083; *P*=0.122)	1.000 (reference)	1.768 (0.913–3.421; *P*=0.091)
Yes	1.393 (0.919–2.111; *P*=0.118)	0.975 (0.646–1.473; *P*=0.906)	2.104 (0.820–5.398; *P*=0.122)	2.309 (1.181–4.516; *P*=0.014)
Drinking status				
No	1.000 (reference)	0.707 (0.518–0.965; *P*=0.029)	1.000 (reference)	1.107 (0.647–1.894; *P*=0.710)
Yes	1.110 (0.622-1.983; *P*=0.724)	0.878 (0.518–0.965; *P*=0.650)	0.286 (0.076–1.081; *P*=0.065)	1.500 (0.783–2.872; *P*=0.221)

In Table 8, patients with advanced age (≥59.5 years) (OR, 95% CI: 1.867 (1.258–2.771), *P*=0.002) and high FBG (≥5.445) (OR, 95% CI: 4.247 (2.534–7.118), *P*<0.001) with the CC genotype of rs3798343 appear to be at risk for CAD. In subjects with the GG+GC genotype, being female (OR, 95% CI: 0.352 (0.226–0.547), *P*<0.001), having low HDL-C (OR, 95% CI: 0.503 (0.322–0.787), *P*=0.003), no smoking (OR, 95% CI: 0.647 (0.441–0.947), *P*=0.025) and not drinking (OR, 95% CI: 0.638 (0.466–0.873), *P*=0.005) serve as protective factors against CAD. Additionally, females (CC: OR, 95% CI: 0.490 (0.318–0.755), *P*=0.001; GG+GA: OR, 95% CI: 0.352 (0.226–0.547), *P*<0.001) and subjects with high HDL-C (HDL-C ≥ 1.325) (CC: OR, 95% CI: 0.534 (0.357–0.799), *P*=0.002; GG+GA: OR, 95% CI: 0.389 (0.254–0.595), *P*<0.001) with either the CC genotype or the GG+GC genotype are less susceptible to CAD. In the recessive model, subjects of advanced age (≥59.5 years) with either the GG genotype (OR, 95% CI: 4.267 (1.366–13.323), *P*=0.013) or the CG+CC genotype (OR, 95% CI: 3.004 (1.439–6.271), *P*=0.003) appear to be at risk for CAD. Patients with elevated FBG (≥5.445) (OR, 95% CI: 3.403 (1.706–6.789), *P*=0.001), low HDL-C (<1.325) (OR, 95% CI: 2.170 (1.000–4.710), *P*=0.050) and smoking habits (OR, 95% CI: 2.098 (1.027–4.284), *P*=0.042) with the CG+CC genotype of rs3798343 are more susceptible to CAD.

**Table 8:** CAD incidence by interactions with environmental factors such as age, gender, smoking status, drinking status, the serum level of HDL-C and FBG

Characteristics	Dominant model of rs3798343 [OR (95% CI); *P-*value]		Dominant model of rs3798343 [OR (95% CI); *P*-value]	
	CC	CG+GG	GG	CG+CC
Age				
<59.5	1.000 (reference)	0.743 (0.497–1.111; *P*=0.148)	1.000 (reference)	1.967 (0.943–4.103; *P*=0.071)
≥59.5	1.867 (1.258–2.771; *P*=0.002)	1.008 (0.667–1.524; *P*=0.969)	4.267 (1.366–13.323; *P*=0.013)	3.004 (1.439–6.271; *P*=0.003)
FBG				
<5.445	1.000 (reference)	0.709 (0.502–1.001; *P*=0.051)	1.000 (reference)	1.163 (0.609–2.218; *P*=0.647)
≥5.445	4.247 (2.534–7.118; *P*<0.001)	1.499 (0.964–2.331; *P*=0.073)	2.125 (0.648–6.968; *P*=0.214)	3.403 (1.706–6.789; *P*=0.001)
HDL-C				
<1.325	1.000 (reference)	0.503 (0.322–0.787; *P*=0.003)	1.000 (reference)	2.170 (1.000–4.710; *P*=0.050)
≥1.325	0.534 (0.357–0.799; *P*=0.002)	0.389 (0.254–0.595; *P*<0.001)	1.553 (0.543–4.443; *P*=0.412)	1.280 (0.596–2.749; *P*=0.527)
Gender				
Male	1.000 (reference)	0.616 (0.433–0.876; *P*=0.007)	1.000 (reference)	1.629 (0.841–3.154; *P*=0.148)
Female	0.490 (0.318–0.755; *P*=0.001)	0.352 (0.226–0.547; *P*<0.001)	1.071 (0.351–3.275; *P*=0.904)	0.786 (0.394–1.568; *P*=0.495)
Smoking status				
No	1.000 (reference)	0.647 (0.441–0.947; *P*=0.025)	1.000 (reference)	1.606 (0.793–3.250; *P*=0.188)
Yes	1.399 (0.941–2.080; *P*=0.097)	0.841 (0.555–1.275; *P*=0.415)	2.256 (0.755–6.743; *P*=0.145)	2.098 (1.027–4.284; *P*=0.042)
Drinking status				
No	1.000 (reference)	0.638 (0.466–0.873; *P*=0.005)	1.000 (reference)	1.139 (0.631–2.057; *P*=0.666)
Yes	1.272 (0.722–2.241; *P*=0.405)	0.697 (0.394–1.234; *P*=0.215)	0.514 (0.115–2.300; *P*=0.384)	1.411 (0.709–2.806; *P*=0.327)

### Haplotype analysis of the SNP sites and CAD

**Figure 1:**
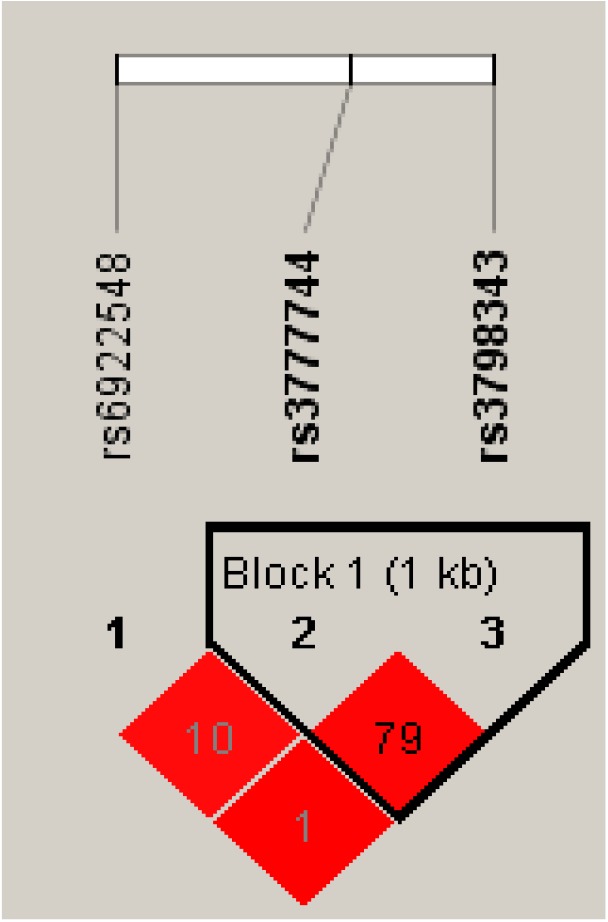
The D′ value of the block formed by rs3777744 and rs3798343.

**Figure 2:**
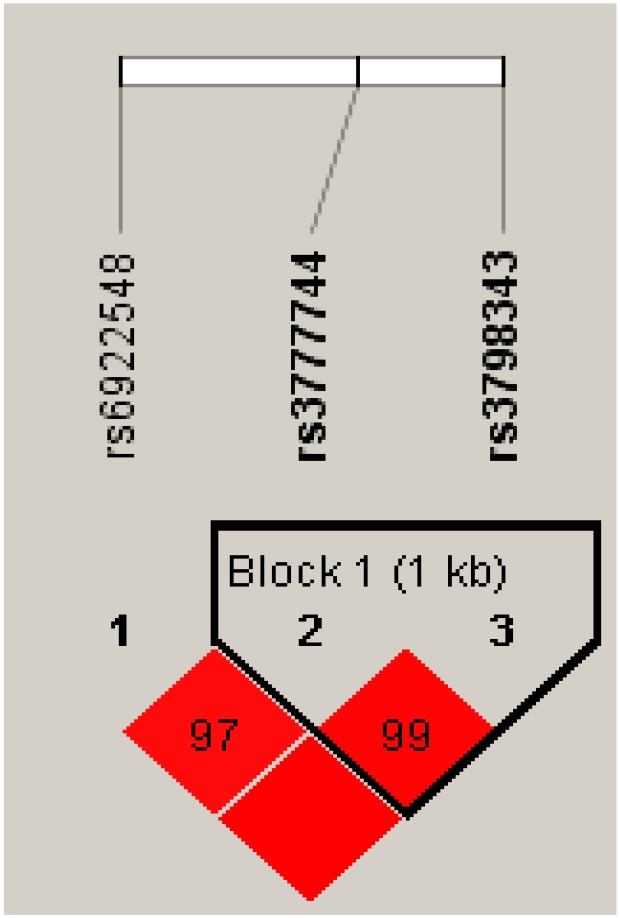
The R-square value of the block formed by rs3777744 and rs3798343

Strong LD can be found between the rs3777744 and rs3798343 genotypes in normal control subjects and CAD patients (r^2^ = 0.79, D′ = 0.99) (Figures 1 and 2). Furthermore, haplotype analyses, including those of the two SNPs and the associations between the different haplotypes and the risk of CAD, have also been carried out. The primary haplotypes are presented in Table 9. The A-C haplotypes were linked with an increased risk of CAD (OR, 95% CI: 1.321 (1.060–1.647), *P*=0.013), and a decreased risk was associated with the G-G haplotypes (OR, 95% CI: 0.714 (0.567–0.849), *P*=0.004).

**Table 9:** Haplotype frequencies of the twp SNPs (rs3777744 and rs3798343) and the risk of CAD

Haploview	Control frequency	CAD frequency	OR (95% CI)	Chi-square	*P*-value
A-C	0.675	0.733	1.321 (1.060–1.647)	6.176	0.013
G-G	0.284	0.221	0.714 (0.567–0.849)	8.223	0.004
G-C	0.037	0.045	1.234 (0.732–2.080)	0.623	0.430

## Discussion

Hyperlipidemia is a major risk factor for AS and CAD [15,16]. Abnormal fatty acid metabolism was found to be related to the severity of ischemic heart disease (IHD) [17]. PPARD is a protein-coding RNA located in chromosome 6 with a length of 85633 bp, and studies show that PPARD plays a significant role in lipid metabolism and oxidation regulation in endothelial cells, vascular smooth muscle cells, and cardiomyocytes [9,18]. In animal experiments with goats, PPARD was found to work as a key transcription factor, together with PPARG, that participates in multiple physiological processes to facilitate lipid secretion and catabolism of fatty acids in mammary epithelial cells [19,20].

Activated PPARD affecting the plasma lipid profile has been reported in humans and animals. GW501516, the PPARD agonist, has been shown to up-regulate the expression of ABCA1 in human monocytic cell lines and increase high-density lipoprotein cholesterol (HDL-C) in monkeys [21]. In another previous study, subjects with low plasma HDL-C concentrations (<1.6 mmol/l) were given different concentrations of GW501516 resulting in the improvement of lipid metabolism [22]. In our study, in the gene–environment interaction analysis, HDL-C played a potential role in the prevalence of CAD as it interacted with different genotypes.

The association between diabetes and CAD has been well established by numerous studies. It is well known that hyperglycemia is a risk factor for coronary heart disease [23]. The pathogenesis of insulin resistance is caused by many factors, including ectopic fat accumulation, increased glucose output, and decreased glucose utilization. Metabolism of glucose is regulated by the liver and skeletal muscle, where PPARD is highly expressed [24,25]. The activation of PPARD strengthens muscular fatty acid oxidation and oxidative phosphorylation, and the oxidative capacity of muscle is positively correlated with systemic insulin sensitivity, meaning that PPARD has a beneficial effect on insulin sensitivity *in vivo* [23]. In our own research, the interaction between elevated FBG and genotype increases the prevalence of CAD, which confirms that hyperglycemia interaction with genotype is a risk factor for CAD.

Recently, SNPs analysis was conducted to explore the molecular mechanisms by which genetic predispositions impact diseases.

Several SNPs in PPARD, including rs2016520 and rs9794, were found to be associated with CVDs [1,7]. In addition, the PPARD SNP rs2016520 (+294C) seemed to be significantly associated with cholesterol metabolism and the risk of CAD [11,26].

To further investigate the relationship between PPARD and CAD, several SNPs including rs3777744, rs3798343, and rs6922548, were selected for the present study.

In a cohort of 880 consecutively sampled patients (609 cases and 271 controls), we found that the distribution of CAD differed between the rs3777744 and rs3798343 polymorphisms. The GG genotype (wild-type) of PPARD rs3777744 decreased the risk of CAD by 27.6% (AOR, 95% CI: 0.724, 0.532–0.985; P*=0.04), and in a dominant model, the GG+GA genotype decreased the risk of CAD by 28.6% (AOR, 95% CI: 0.714, 0.534–0.954; *P*=0.023). Additionally, the GG genotype of PPARD rs3798343 decreased the risk of CAD by 39.4% (AOR, 95% CI: 0.606, 0.445–0.825; P*=0.001), and the GG+GA genotype decreased the risk of CAD by 38.3% (AOR, 95% CI: 0.617, 0.460–0.826; P*=0.001). We found that high serum glucose levels and low high-density lipoprotein cholesterol levels were shown to increase the risk of CAD, and the effect was more pronounced when the serum indicators interacted with genetic factors. A sequential haplotype analysis was conducted and linkage disequilibrium was found between rs3777744 and rs3798343 (r^2^ = 0.79, D′ = 0.99) (Figures 1 and 2). This is the first study on the relationship amongst rs3777744, rs3798343, and CAD.

**Figure 3:**
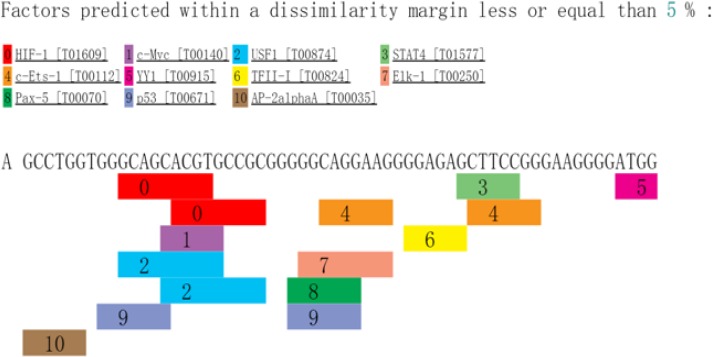
The possible related transcription factors binding to the rs3777744 SETD2, SET domain containing 2; MYC, MYC proto-oncogene, bHLH transcription factor; USF1, upstream transcription factor 1; STAT4, signal transducer and activator of transcription 4; CETS1, ETS proto-oncogene 1, transcription factor; YY1, YY1 transcription factor; GTF2I, general transcription factor Iii; ELK1, ELK1, ETS transcription factor; PAX5, paired box 5; P53, protein p53; AP2A, Transcription factor AP-2α.

**Figure 4:**
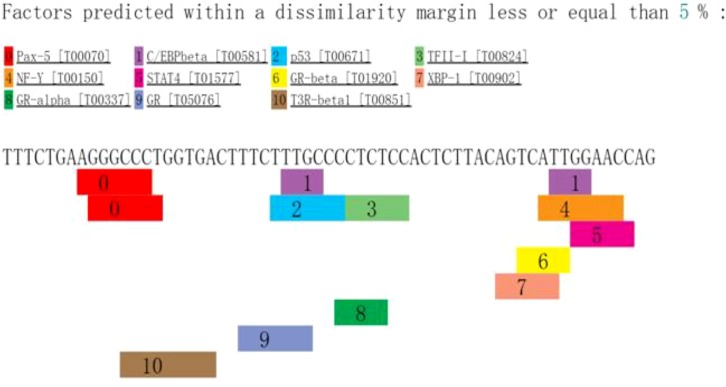
The possible related transcription factors binding to the rs3798343 PAX5, paired box 5; CEBPB, CCAAT enhancer binding protein β; P53, protein p53; GTF2I: general transcription factor Iii; NFY(A/B/C), Official Symbol nuclear transcription factor Y subunit α/β/γ; STAT4: signal transducer and activator of transcription 4; GR-BETA: Official Symbol NR3C1 nuclear receptor subfamily 3 group C member 1-β; XBP1: X-box binding protein 1; GR-ALPHA, Official Symbol NR3C1 nuclear receptor subfamily 3 group C member 1-α; NR3C1 nuclear receptor subfamily 3 group C member 1; THRA, thyroid hormone receptor α.

**Figure 5:**
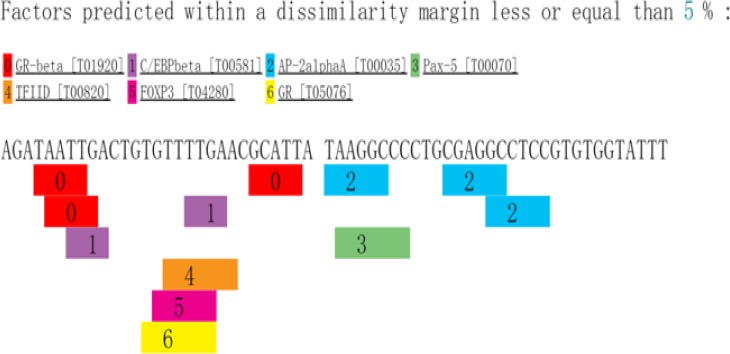
The possible related transcription factors binding to the rs6922548 GR-BETA, Official Symbol NR3C1 nuclear receptor subfamily 3 group C member 1-β; CEBPB: CCAAT enhancer binding protein β; AP2A, Transcription factor AP-2 α; PAX5, paired box 5; TBP, TATA-box binding protein; FOXP3, forkhead box P3; NR3C1, nuclear receptor subfamily 3 group C member 1.

The intron variants rs3777744 and rs3798343 occur within an intron located in the gene for PPARD. Transcription variants within an intron have numerous possibilities for regulating genes, such as affecting alternative splicing of the mRNA and enhancing the expression of multiple genes. Although a significant association was found between CAD and the PPARD polymorphisms in the present study, more evidence is needed to conclude that SNPs are functional. SNPs can be associated in some populations due to correlations with other SNPs that actually impact the regulation of the gene. The linkage disequilibrium was found between rs3777744 and rs3798343 (r^2^ = 0.79, D′ = 0.99) in haplotype analysis. Subsequently, a methylated CpG islands prediction was conducted using the USUC database. Areas upstream and downstream (1000 bp) of the studied SNPs were included in the analyzed sequences. However, none of the methylation islands were found in the target sequences. In addition, a transcription factor prediction using the PROMO database was conducted, and the analyzed sequences included the areas upstream and downstream (30 bp) of the three SNPs. The possible related transcription factors that bind to the functional variants are shown in Figures 3–5. The data implied that CETS1, ELK1, PAX5, and P53 bind to rs3777744, P53 binds to rs3798343, and AP2A and PAX5 specifically bind to rs6922548. Additional transcription factors were predicted to bind to the analyzed sequences (Figures 3–5). P53 (also called TP53, tumor protein P53) is a widely studied protein-coding gene that is significantly related to neoplasms. In addition, P53, along with its functional polymorphism, has been linked to CAD in recent years [27–29].

A subsequent KEGG pathway analysis within the transcription factors was conducted. TNF (*P*=0.170) and ErbB (*P*=0.136) signaling pathways, which have been found to play key roles in CAD occurrence and development [30–32], were suggested as critical regulatory factors.

In the present study, rs3777744 and rs3798343 influenced the prevalence of CAD. We hypothesized that these two polymorphisms participated in the occurrence and development of CAD by targetting P53, and the TNF and ErbB signaling pathways acted as metabolic regulatory factors in CAD under the influence of the variants.

However, this pathway prediction is unreliable due to the small number of predicted transcription factors. Further studies are needed to explore the pathological mechanism.

## Limitations

First, the allele distribution of rs6922548 is consistent with the Hardy–Weinberg Equilibrium, but the genotype distribution of rs6922548 shows no difference in between the CAD and control groups, so a larger sample size is needed to study the relationship between CAD and rs6922548. Second, the results of the analysis in this work reveal only the statistical correlation between PPARD polymorphisms and CAD, so a professional analysis of the data should be conducted for more convincing results. Third, a molecular mechanism study is needed to explore the role that PPARD plays in CAD.

## Conclusion

In conclusion, there is a significant association between the PPARD rs3777744 G-allele, rs3798343 G-allele, and a decreased risk for CAD. Furthermore, we found that high HDL-C serum levels, low glucose levels, and genotypes interact, ultimately decreasing the risk for CAD.

## Abbreviations

AOR: adjusted odds ratio
Apo-A: apolipoprotein A
Apo-B: apolipoprotein B
AS: atherosclerosis
CAD: coronary artery disease
CI: confidence interval
CVD: cardiovascular disease
FBG: fasting blood glucose
HDL-C: fasting high-density lipoprotein cholesterol
LDL-C: fasting low-density lipoprotein cholesterol
MAF: minor allele frequency
OR: odds ratio
PPAR: peroxisome proliferator-activated receptor
PPARA: PPAR α
PPARD: PPAR Δ
PPARG: PPAR γ
ROC: receiver operating characteristic
SAP: shrimp alkaline phosphatase
SNP: single nucleotide polymorphism
TC: total cholesterol
TG: triglyceride
